# Treatment Efficacy of MEDI3902 in Pseudomonas aeruginosa Bloodstream Infection and Acute Pneumonia Rabbit Models

**DOI:** 10.1128/AAC.00710-19

**Published:** 2019-07-25

**Authors:** Hoan N. Le, Vuvi G. Tran, Trang T. T. Vu, Emmanuelle Gras, Vien T. M. Le, Marcos Gabriel Pinheiro, Fábio Aguiar-Alves, Erika Schneider-Smith, Henry Clay Carter, Bret R. Sellman, C. Kendall Stover, Antonio DiGiandomenico, Binh An Diep

**Affiliations:** a Division of HIV, Infectious Diseases, and Global Medicine, Department of Medicine, University of California, San Francisco, San Francisco, California, USA; b François Rabelais University, Tours, France; c Pathology Program, Fluminense Federal University, Rio de Janeiro, Brazil; d Microbial Sciences, AstraZeneca, Gaithersburg, Maryland, USA

**Keywords:** *Pseudomonas aeruginosa*, bloodstream infections, immunotherapy, pneumonia

## Abstract

Pseudomonas aeruginosa is a challenge for clinicians due to increasing drug resistance and dwindling treatment options. We report on the activity of MEDI3902, an antibody targeting type 3 secretion protein PcrV and Psl exopolysaccharide, in rabbit bloodstream and lung infection models. MEDI3902 prophylaxis or treatment was protective in both acute models and exhibited enhanced activity when combined with a subtherapeutic dose of meropenem. These findings further support MEDI3902 for the prevention or treatment of serious P. aeruginosa infections.

## Abstract (precis)

Pseudomonas aeruginosa is a challenge for clinicians due to increasing drug resistance and dwindling treatment options. We report on the activity of MEDI3902, an antibody targeting type 3 secretion protein PcrV and Psl exopolysaccharide, in rabbit bloodstream and lung infection models. MEDI3902 prophylaxis or treatment was protective in both acute models and exhibited enhanced activity when combined with a subtherapeutic dose of meropenem.

## TEXT

Pseudomonas aeruginosa is the second most common cause of nosocomial pneumonia, health care-associated pneumonia, and ventilator-associated pneumonia and the seventh most common cause of nosocomial bloodstream infections (1). These infections are associated with significant mortality and morbidity, increased intensive care unit and hospital length of stay, and substantial economic burden (2). Extensively drug-resistant (XDR) and pandrug-resistant (PDR) P. aeruginosa infections have been reported on multiple continents and are associated with higher mortality rates (3–6). Therefore, novel therapeutics targeting this problematic pathogen are urgently needed to address this significant unmet medical need.

MEDI3902 is a bispecific monoclonal antibody (MAb) targeting the type 3 secretion (T3S) protein PcrV and the Psl exopolysaccharide and is currently under clinical evaluation for the prevention of pneumonia in ventilated patients (Effort to Prevent Nosocomial Pneumonia Caused by Pseudomonas aeruginosa in Mechanically Ventilated Subjects [EVADE] study, ClinicalTrials.gov NCT02696902). Both virulence determinants have been implicated in immune evasion (7–9) MEDI3902 mediates three distinct mechanisms of action, as follows: (i) binding to PcrV prevents T3S injectisome-mediated cytotoxicity and damage to host cells (10), (ii) binding to Psl promotes opsonophagocytic killing of P. aeruginosa by host effector cells, and (iii) binding to Psl inhibits the bacterium’s ability to attach to host epithelial cells (11, 12). Mouse models have been used extensively to demonstrate preclinical protective efficacy, although it should be noted that this animal species is known to be extremely resistant to the effects of Gram-negative lipopolysaccharide (LPS) (13). In contrast to rodents and monkeys, rabbits are exquisitely similar to humans and chimpanzees in their susceptibilities to LPS (13).

**FIG 1:**
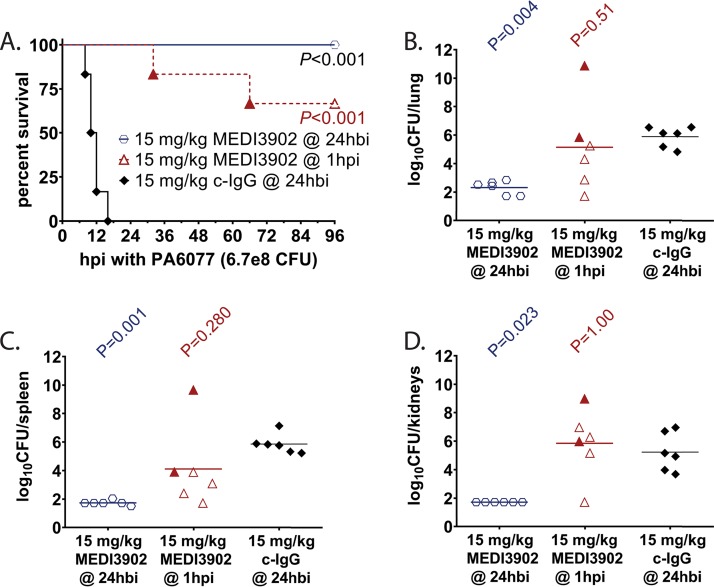
MEDI3902 prophylaxis and treatment improves survival outcome in a rabbit bloodstream infection model. (A to D) Comparisons of Kaplan-Meier survival curves (A), log_10_ CFU/lung (B), log_10_ CFU/spleen (C), and log_10_ CFU/kidneys (D) for rabbits administered intravenously with 15 mg/kg MEDI3902 at 24 h before infection (hbi) (*n* = 6), 15 mg/kg MEDI3902 at 1 h postinfection (*n* = 6), and 15 mg/kg control IgG at 24 h before infection (*n* = 6) with strain 6077. A one-sided log rank (Mantel-Cox) test was used to evaluate survival of control IgG- versus MEDI390-treated animals. Bacterial densities for animals pretreated with 15 mg/kg control IgG were compared to those pretreated or treated with 15 mg/kg MEDI3902 by nonparametric one-way analysis of variance (ANOVA) with Kruskal-Wallis test, followed by Dunn’s multiple-comparison test. Filled symbols represent data from dead animals, and open symbols represent data from surviving animals that were euthanized at 96 h postinfection.

Therefore, we evaluated MEDI3902 efficacy in a rabbit model of bloodstream infection. Rabbits were randomized for intravenous administration with (i) 15 mg/kg of body weight control IgG at 24 h before infection, (ii) 15 mg/kg MEDI3902 at 24 h before infection, and (iii) 15 mg/kg MEDI3902 at 1 h postinfection and then challenged by intravenous injection of the cytotoxic P. aeruginosa strain 6077 contained within 1.5 ml lactated Ringer’s solution at 4.4e8 CFU/ml. Rabbits were monitored every 2 h for the first 36 h postinfection and then at least three times daily thereafter. All survivors were euthanized at 96 h postinfection. Overall survival rates were 0% (0/6) for rabbits administered control IgG, 100% (6/6) for animals receiving MEDI3902 24 h before infection (*P* < 0.001 versus control IgG group by log rank test), and 67% (4/6) for those administered MEDI3902 at 1 h postinfection (*P* < 0.001 versus control IgG by log rank test) (Fig. 1A). Bacterial counts from homogenized organs were significantly reduced in vital organs of rabbits administered with MEDI3902 at 24 h before infection (Kruskal-Wallis test with Dunn’s multiple-comparison test for each MEDI3902 dosage versus control IgG), but some loss in protection was observed when delivered at 1 h postinfection (Fig. 1B to D). The lower limit of bacterial detection in our organ burden model was 1.72 log CFU/organ.

**FIG 2:**
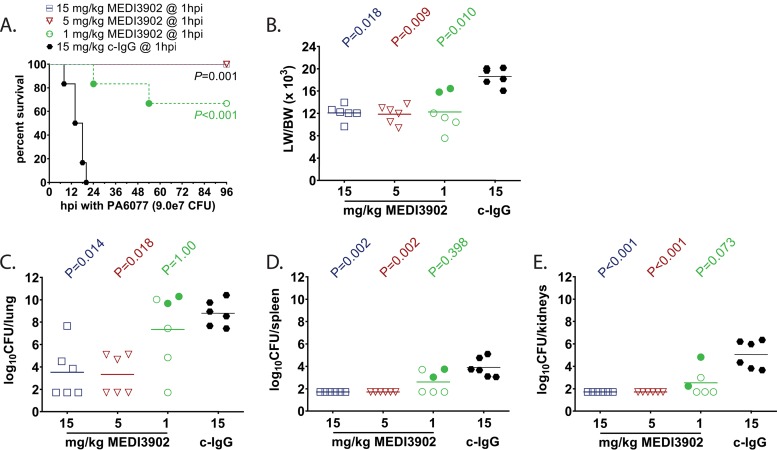
Treatment with MEDI3902 improves survival outcomes in a dose-dependent manner in a rabbit acute pneumonia model. (A to E) Comparisons of Kaplan-Meier survival curves (A), lung weight-to-body weight (LW/BW ×10^3^) ratio (B), log_10_ CFU/lung (C), log_10_ CFU/spleen (D), and log_10_ CFU/kidneys (E) for rabbits intravenously administered 15 mg/kg MEDI3902 (*n* = 6), 5 mg/kg MEDI3902 (*n* = 6), 1 mg/kg MEDI3902 (*n* = 6), or 15 mg/kg control IgG (*n* = 6) at 1 h postinfection (hpi). A one-sided log rank (Mantel-Cox) test was used to evaluate survival. LW/BW and bacterial densities for animals treated at 1 h postinfection with control IgG were compared to those treated with different concentrations of MEDI3902 by nonparametric one-way ANOVA with Kruskal-Wallis test, followed by Dunn’s multiple-comparison test. Filled symbols represent data from dead animals, and open symbols represent data from surviving animals that were euthanized at 96 h postinfection.

For treatment studies in the rabbit model of pneumonia, rabbits were randomized for intravenous treatment with MEDI3902 at 1, 5, and 15 mg/kg or control IgG (15 mg/kg) at 1 h postinfection. To induce pneumonia, a 1.5-ml inoculum containing 9e7 CFU/ml of strain 6077 was delivered directly into the lungs of anesthetized rabbits through a pediatric endotracheal tube (outside diameter of 2.5 mm) and then immediately removed after instillation of bacterial inoculum. Rabbits were monitored every 2 h for the first 36 h after infection and three times daily thereafter. Rabbits that became severely ill as defined by signs of pulmonary dysfunction (respiration rate of >75 breaths/minute, cyanosis, and cough) were euthanized for humane reasons and scored as nonsurvivors. All survivors were euthanized at 96 h postinfection. Overall survival rates were 0% (0/6) for rabbits treated with control IgG, compared to 100% (6/6) for those treated with MEDI3902 at 15 or 5 mg/kg and 66% (4/6) for those treated with 1 mg/kg (*P* < 0.001 for treatment with various doses of MEDI3902 compared to control IgG group by log rank test; Fig. 2A). The lung weight-to-body weight (LW/BW) ratio, a quantitative measurement of pulmonary edema and a surrogate marker of the severity of acute lung injury, was significantly reduced for all rabbits treated with MEDI3902 compared to control IgG-treated rabbits (Kruskal-Wallis test with Dunn’s multiple-comparison test for each MEDI3902 dosage versus control IgG; Fig. 2B). Bacterial counts in the lungs, spleen, and kidneys were significantly reduced in rabbits treated with MEDI3902 at 15 mg/kg and at 5 mg/kg compared to those in control IgG-treated rabbits (Kruskal-Wallis test with Dunn’s multiple-comparison test for each MEDI3902 dosage versus control IgG) (Fig. 2C to E).

**FIG 3:**
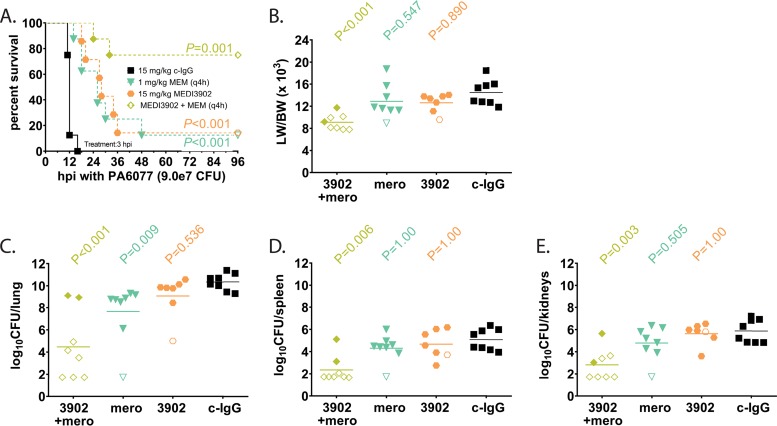
Adjunctive therapy of MEDI3902 with a subtherapeutic dose of meropenem in a rabbit acute pneumonia model. (A to E) Comparisons of Kaplan-Meier survival curves (A), lung weight-to-body weight (LW/BW ×10^3^) ratio (B), log_10_ CFU/lung (C), log_10_ CFU/spleen (D), and log_10_ CFU/kidneys (E) for rabbits administered intravenously with (i) 15 mg/kg MEDI3902 (*n* = 7), (ii) 1 mg/kg meropenem i.v. q4h (*n* = 8), (iii) a 15 mg/kg MEDI3902 plus 1 mg/kg meropenem i.v. q4h (*n* = 8) combination, or (iv) 15 mg/kg of control IgG (*n* = 8) at 3 h postinfection with strain 6077. A one-sided log rank (Mantel-Cox) test was used to evaluate survival. LW/BW and bacterial densities for animals pretreated with 15 mg/kg control IgG were compared to those treated with MEDI3902 alone, meropenem alone, or the combination by nonparametric one-way ANOVA with Kruskal-Wallis test, followed by Dunn’s multiple-comparison test. Filled symbols represent data from dead animals, and open symbols represent data from surviving animals that were euthanized at 96 h postinfection.

We next sought to evaluate the activity of MEDI3902 used adjunctively with meropenem in the rabbit model of pneumonia. For these studies, subprotective dosages of meropenem to simulate drug resistance and MEDI3902 were identified. Intravenous (i.v.) treatment with meropenem starting at 3 h postinfection with 75 mg/kg and 10 mg/kg meropenem every 4 hours (q4h) protected virtually all treated rabbits against death in the rabbit acute pneumonia model (data not shown), whereas 1 mg/kg of meropenem i.v. q4h was nonprotective. We also found in preliminary studies that intravenous treatment of 15 mg/kg MEDI3902 at 3 h postinfection was also nonprotective. We next sought to determine whether adjunctive therapy with MEDI3902 (15 mg/kg) and meropenem (1 mg/kg q4h) conferred protection against acute pneumonia. Compared to control IgG-treated rabbits that all rapidly succumbed to infection, intravenous treatment with 15 mg/kg MEDI3902 or 1 mg/kg meropenem q4h alone significantly delayed the time to death, but ultimately, the vast majority of these animals died of profound acute respiratory failure, as evidenced from the LW/BW (×10^3^) ratio of >10 (survival analysis performed by log rank test; LW/BW analysis performed by Kruskal-Wallis test with Dunn’s multiple-comparison test for each MEDI3902 dosage versus control IgG) (Fig. 3A and B). In contrast, the treatment combination of 15 mg/kg MEDI3902 and 1 mg/kg meropenem q4h significantly protected against death (log rank test) (Fig. 3A) by protecting against acute lung injury (Fig. 3B) and reducing bacterial counts in the lungs, spleen, and kidneys (Kruskal-Wallis test with Dunn’s multiple-comparison test for each MEDI3902 dosage versus control IgG) (Fig. 3C to E).

In conclusion, our results confirm the protective activity of MEDI3902 used preclinically in prophylaxis and treatment regimens, as well as when used adjunctively with antibiotic.
